# Mechanobiology of Colorectal Cancer

**DOI:** 10.3390/cancers14081945

**Published:** 2022-04-12

**Authors:** Maria Manuela Brás, Susana R. Sousa, Fátima Carneiro, Manfred Radmacher, Pedro L. Granja

**Affiliations:** 1 Instituto de Investigação e Inovação em Saúde (i3S), Universidade do Porto, 4200-135 Porto, Portugal; mbras@i3s.up.pt (M.M.B.); ssousa@ineb.up.pt (S.R.S.); fcarneiro@ipatimup.pt (F.C.); pgranja@i3s.up.pt (P.L.G.); 2 Instituto de Engenharia Biomédica (INEB), Universidade do Porto, 4200-135 Porto, Portugal; 3 Faculdade de Engenharia da Universidade do Porto (FEUP), 4200-465 Porto, Portugal; 4 Instituto Superior de Engenharia do Porto (ISEP), Instituto Politécnico do Porto (IPP), 4200-072 Porto, Portugal; 5 Instituto de Patologia e Imunologia Molecular da Universidade do Porto (IPATIMUP), 4200-465 Porto, Portugal; 6 Serviço de Patologia, Centro Hospitalar Universitário de São João (CHUSJ), 4200-319 Porto, Portugal; 7 Faculdade de Medicina da Universidade do Porto (FMUP), 4200-319 Porto, Portugal; 8 Institute for Biophysics, University of Bremen, 28334 Bremen, Germany

**Keywords:** colorectal cancer, biophysical cues, biochemical pathways, mechanobiology, atomic force microscopy

## Abstract

**Simple Summary:**

It is well documented that colorectal cancer (CRC) is the third most common cancer type, responsible for high mortality in developed countries, resulting in a high socio-economic impact. Several biochemical and gene expression pathways explaining the manifestation of this cancer in humans have already been identified. However, explanations for some of the related biophysical mechanisms and their influence on CRC remain elusive. In CRC, biophysics and medical research have already revealed the importance of studying the effects of the stiffness and viscoelasticity of the substrate on cells, as well as the effect of the shear stress of blood and lymphatic vessels on the behavior of cells and tissues. A deeper understanding of the relationship between the biophysical cues and biochemical signals could be advantageous to develop new diagnostic techniques and therapeutic strategies. Being a disease with a high mortality rate, it becomes crucial to dedicate efforts to finding effective, alternative therapeutic strategies.

**Abstract:**

In this review, the mechanobiology of colorectal cancer (CRC) are discussed. Mechanotransduction of CRC is addressed considering the relationship of several biophysical cues and biochemical pathways. Mechanobiology is focused on considering how it may influence epithelial cells in terms of motility, morphometric changes, intravasation, circulation, extravasation, and metastization in CRC development. The roles of the tumor microenvironment, ECM, and stroma are also discussed, taking into account the influence of alterations and surface modifications on mechanical properties and their impact on epithelial cells and CRC progression. The role of cancer-associated fibroblasts and the impact of flow shear stress is addressed in terms of how it affects CRC metastization. Finally, some insights concerning how the knowledge of biophysical mechanisms may contribute to the development of new therapeutic strategies and targeting molecules and how mechanical changes of the microenvironment play a role in CRC disease are presented.

## 1. A Brief Introduction to Colorectal Cancer (CRC)

Sung et al. presented the cancer incidence and mortality provided by the International Agency for Research on Cancer (IARC) at GLOBOCAN 2020. Worldwide, breast cancers present the highest number of new cases (11.7%), followed by lung (11.4%) and CRC (10.0%). Lung cancer was the leading cause of cancer death (18%), followed by CRC (10%). In summary, CRC is the third in terms of incidence but the second in terms of mortality [1,2].

The risk of CRC has been growing over the past few years due to environmental factors, a sedentary lifestyle, and diet [3]. Shussman and Wexner referred that multiple, cumulative genetic alterations lead to the formation of a neoplastic process, normally explained taking into consideration molecular biology and biochemical phenomena, and frequently not through biophysics, in which the forces exerted by the cells and the ECM are translated into biochemical signals (mechanotransduction) [4]. A deeper understanding of biophysical mechanisms behind biochemical pathways can lead to the development of new therapeutic strategies. For that purpose, it is important to understand the influence of several physical cues, such as: (i) internal forces of the cells; (ii) forces produced by the neighboring cells; (iii) forces coming from interstitial spaces (such as mechanical tension, compression, and hydrostatic pressure); (iv) forces resulting from the alterations of ECM stiffness; (v) shear stress (the parallel force per unit area applied to cell walls) from the fluid flow (such as blood); and (vi) forces from adhesion molecules [5]. Understanding the role of biophysical cues makes it possible to evaluate which biomechanical response will be activated. Each response will induce changes in CRC cell behavior (increased motility, shape changes, stiffness changes) which can promote the CRC cell motility, migration, invasion, extravasation, and metastization. The origin of adenocarcinomas, as well as the large intestine tissue with its several different cell types and functions has already been well described in the literature [3,4,6,7,8,9].

Not every CRC shares similar driving mutations, making it extremely difficult to design a general molecular therapy [10]. Surgery is the initial approach in terms of treatment if an early diagnosis is performed. However, if it has already been metastasized, this approach is not effective anymore. These patients also develop drug resistance, and the recurrence of cancer is very common [11].

### 1.1. From Crypt Dysfunction to Polyp Formation

**Figure 1:**
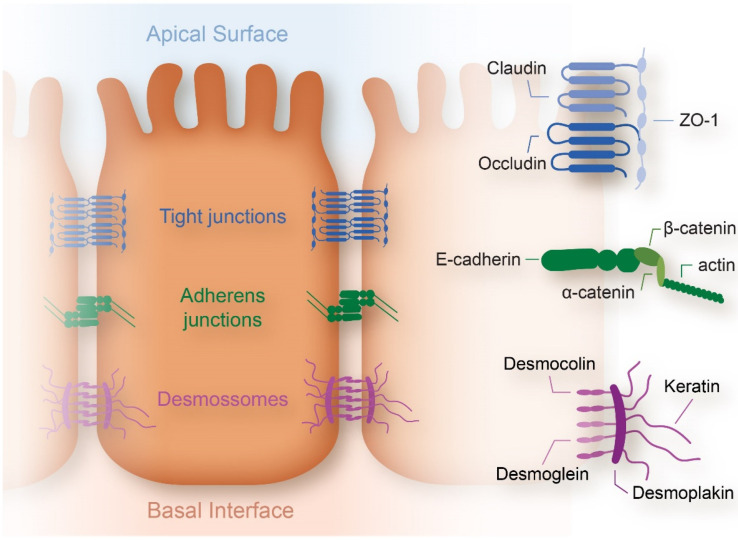
Epithelial cells at human colon intestine, with the adherens junctions, tight junctions and desmosomes. The adherens junctions are complexes that assemble and disassemble, allowing cells to respond to forces, biomechanical signals, and structural changes in their microenvironment. The tight junctions (occludins and claudins) are intercellular adhesion complexes, that constitute a paracellular diffusion semipermeable barrier, being charge and size selective. The desmosomes are strong cell-cell adhesion molecules, mainly present in tissues that sense intense mechanical stress. Adapted from Balda et al. [14].

CRC begins at the epithelial cells, starting with adenoma (polyps with potential for carcinogenesis) and evolving to adenocarcinoma, existing at the mucosa layer that lines the colon and rectum [3,12,13]. The epithelial cells are connected between them through tight junctions (claudin and occludin) which, in turn, are connected to the cytoskeleton (myosin and F-actin filaments) through the myosin light-chain kinase (MLCK) and zonula occludins (ZO), such as ZO-1 [14]. The function and constitution of tight junctions have also been previously well described by Balda et al. [14]. The adherens junctions are formed by E-cadherin, β-catenin, α-catenin 1 and δ1-catenin (also known as p120 catenin), and the desmosomes, formed by the desmoglein and desmocolin. These two proteins bind to keratin through desmoplaquin, and then keratin binds to the cell nucleus (Figure 1). The expression dysregulation of some tight junctions, such as ZO-1 and ZO-2, may lead to some types of cancer, such as breast cancer [14,15].

Lechuga et al. reported recently that epithelial junctions are exposed to constant protrusive, tensile, and contractile forces which are generated by two cytoskeletal motors: actin filament polymerization and myosin II-dependent contractility [16]. The activity of these motors is controlled by biochemical and mechanical properties of the environment. Altered mechanical properties of tissues in different diseases should not be assumed to be modeled by our understanding of the junction-associated actomyosin. Ex vivo primary epithelial cell organoids and animal models with epithelial-specific knockouts of different actin regulators should be introduced as innovative models, providing novel critical insights to the understanding of the roles and mechanisms of cytoskeletal regulation of epithelial barriers during mucosal inflammation [16].

Cellular attachment to the underlying basal lamina induces proliferation in the epithelium, thereby generating stress in it, causing its buckling, thus inducing a displacement and initiating crypt budding and fission. Although still lined by a single layer of epithelial cells, adenomatous crypts exhibit multiple branching events and are typically elongated and deformed [17,18]. Van Leeuwen et al. explained that an increase in cell proliferation, accompanied by a reduction in cell-cell adhesion, may lead to crypt fission [19].

The buckling of a colorectal crypt is described by models developed for this purpose. One of the models describes a cross-section of a colorectal crypt when it is unfolded and modeled as a beam connected to the tissue by a series of viscoelastic springs. The authors hypothesized that buckling was driven by growth of the epithelium and explored the consequences of altered proliferation and stromal adhesion. They predicted that an increase in proliferation could initiate buckling if there was a sufficiently flexible layer [20]. Different epithelial folding, such as bending (related with myosin differentials), buckling (related with myosin compression), growth, and pumping (related with osmotic pressure), were studied in the framework of the mechanobiology of intestinal organoids [21,22,23,24].

The old, damaged, or no longer needed colon epithelial cells are replaced every 3–5 days by new healthy cells through several events of mechanical functions, such as division, migration, and extrusion [25].

**Figure 2:**
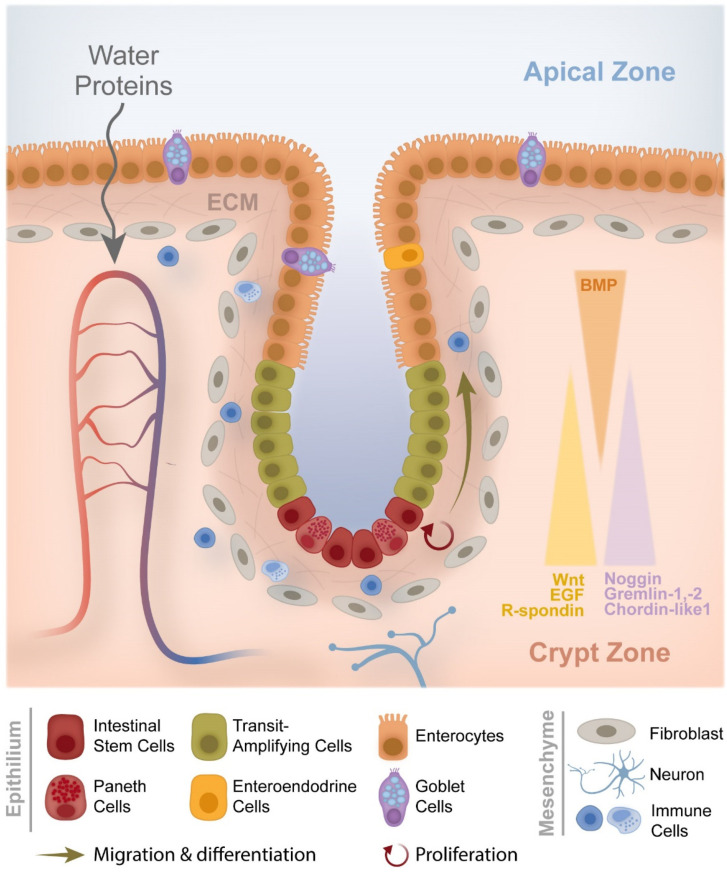
The intestinal stem cells are at the bottom of the crypt. These cells give rise to transit amplifying cells. During their differentiation, these cells migrate along the crypt, reaching the surface. Intestine stem cells regulation, as well as the proliferation and differentiation processes, are mediated by signals which come from the surrounding mesenchymal cells. At the bottom of the crypt, are expressed genes (Wnt, R-spondin, EGF) that regulate the cell cycle, and the intestine stem cells and progenitors increase, despite the genes inducing apoptosis being downregulated. The bone morphogenetic protein (BMP) pathways are expressed on the top of the crypt. In the meantime, BMP antagonists (noggin, gremlin, and chordin-like 1) are expressed, at the bottom of the crypt, by stromal cells. R-spondin is a protein that promotes the canonical Wnt/β-catenin pathway signaling. Adapted from Audrey [6,15,26]. ECM—extracellular matrix; EGF—Epidermal growth factor; Wnt—Wingless and Int-1.

Figure 2 shows the large intestine tissue with its several different cell types and the normal regulation mechanisms of migration and cell differentiation on the crypt.

The growth and division of new cells are usually regulated, although sometimes new cells grow and divide before they are required, thus originating a polyp. These polyps result from a failure or inability in relation to the proliferation, differentiation, or apoptosis of the epithelial cells at the mucosa. Polyps are classified according to the following parameters: macroscopic appearance (flat or sessile and with a stalk or pedunculated), size, number, anatomic distribution, and histology [4].

### 1.2. CRC Formation and Development

In terms of microscopical anatomy, the colon is composed by different layers, with specific cell types and functions, which are well described by several authors [27,28,29,30].

CRC can be classified according to 11 stages using the tumor, nodes, and metastasis (TNM) system, which is based on three key pieces of information, according to the American Cancer Society: tumor (extent of the tumor), nodes (assessment if cancer spread to nearby lymph nodes), and metastasis (assessment if cancer spread to distant lymph nodes or distant organs, such as the liver or lungs) [31]. Cancer cells metastasize to the lymph nodes via lymphatic vessels and to distant organs (liver, lungs, etc.) via blood vessels [3].

The beginning of CRC is related to a deficiency in cell migration from the crypt, which depends on the adenomatous polyposis coli (APC) protein. If there is a loss in this protein, then cell migration is stopped, leading to the accumulation of cells in the amplified zone of the crypt. The amount of these cells is then increased, all of them accumulating mutations, thus resulting in the formation of a tumor. The epithelium is very sensitive to mutation and carcinogenesis, since the cell replication rate in the epithelium of the colon and rectum is high, with a replication rate of 1010 cells every day [32]. In CRC, the epithelial-to-mesenchymal transition (EMT) drives cellular migration, characterized by the acquisition of a mesenchymal phenotype through the dissolution of tight junctions, disruption of apical-basal polarity, and reorganization of the cytoskeletal architecture [33].

## 2. CRC Mechanotransduction

Cancer mechanics is the study of forces existing between tumor cells and the extracellular environment. Sawminathan et al. mentioned the biophysical stimuli that cells sense from the microenvironment. Forces are produced by the neighboring cells and by the confined interstitial spaces, such as tension, compression, and hydrostatic pressure. Alterations in the ECM stiffness and shear stress from fluids (such as blood) are also sensed by the cells. The response to these mechanical stimuli will be different regarding the cell and the tissue type [34]. Mechanotransduction use several ways to respond to biophysical cues: (i) mechanosensors in the cell membrane (transmembrane receptors, growth factor receptors and proteins that mediate cell-cell adhesion and cell-ECM interactions), (ii) mechanosensitive ion channels, (iii) ECM proteins, (iv) cytoskeleton components, and (v) nuclear structures (involving the chromatin) [5]. Proteins, such as α-catenin, β-catenin, integrins, von Willebrand factor (vWf), talin, vinculin, p130 Crk-associated substrate (Cas), focal adhesion kinase (FAK), and Src kinases, are included in the focal adhesions and adherens junctions [5].

Mechanical stimuli also change due to: (i) cell polarity, motility, shape, geometry, topography, and roughness changes [35]; and (ii) ECM stiffness and tissue geometry changes [36]. Mechanical features inform cells which biochemical pathways need to be up-or downregulated to maintain tissue homeostasis [37]. In the next sections, the importance of the nucleus envelope, cytoskeleton, cell membrane, stroma, and ECM will be addressed. Some examples of cell studies will be given to correlate the physical parameters in the CRC cell behavior and impact on mechanical properties, and the influence of the environment, such as the stroma and ECM, will be addressed in cells mechanotransduction.

### 2.1. Contributors for Mechanotransduction

#### 2.1.1. Nuclear Envelope

The main components of a cell are nucleus, cytoplasm, and cellular membrane and they have important functions in cellular biophysics. The main nucleus mechanics components are the nuclear membrane, membrane proteins, lamina, chromatin, and intranuclear proteins. The nuclear membrane protein and the lamin (protein network) form the inner nuclear envelope. The inner nuclear layer and the lamina are continuous. Lamina is divided into lamina A (lamin A and C) and type B lamina. Lamina supports the nucleus and is defined by the shape and size of it. Nuclei that contain more lamin A are stiffer and more resistant to deformation. Lamin binds to chromatin and other intranuclear proteins, forming a nucleoskeleton system, resisting mechanical stress, and being a scaffold in transcriptional regulation. The nucleus is coupled to the cytoskeleton through the nesprin. This binds to lamin A/C and cytoskeleton proteins bind through the linker of nucleoskeleton and cytoskeleton complex (LINC) [38]. This complex is the connection through which the forces from outside the cells are sensed inside the nucleus.

Several studies reported that nuclear membrane proteins are associated with the progression of cancers and thus can be used as biomarkers, such as colon cancer-specific antigen-3 (CCSA-3) for colon cancer [39]. The mesh network of the nuclear lamina provides support to nuclear size and viscoelastic behavior of the nucleus when subjected to external forces [40,41]. For instance, the depletion of lamin B does not cause any change in stiffness but causes additional blebbing in the nucleus [42]. This means that lamin A is the dominant factor in controlling nuclear stiffness [43], which has a value ranging from 0.1 to 10 kPa. The viscosity of the lamina is controlled by lamin A, enabling the nucleus to support the applied force [44]. Changes in nuclear proteins induce malformation in cell division, migration, signaling, and gene expression, such as the overexpression of lamin A. This stimulates the reconstruction of the cytoskeleton via the upregulation of actin-binding protein plastin-3 and downregulation of E-cadherin in colon cancer cells (CCCs), thus resulting in the increase of migration and invasiveness of cancer cells [44]. Pennacchio et al. explained the contribution of the nucleus in mechanotransduction in a comprehensive manner [45].

#### 2.1.2. Cytoskeleton

The cytoskeleton is composed of a network of proteins distributed in the cytoplasm, constituted by the actin microfilaments, intermediate microfilaments, and microtubules. All of them have different mechanical properties, their function being the stabilization of cell morphology and definition of its architecture. Actin remodeling is associated with different cancer phenotypes as well as mechanical property changes. The cytoskeleton is composed by F-actin, a filamentous polymer, composed of G-actin monomers. This polymerization/depolymerization process contributes to cell motility, regulating several actin signaling proteins that constitute part of the oncogenic signaling pathways, which will be discussed in Section 2.4. Usually, epithelial cells have normal differentiation, which is characterized by actin polymerization. Actin depolymerization occurs in the pre-cancerous stages. Cancer progresses in situ to become invasive, co-existing with morphological cell alteration, such as the increase of nuclear-cytoskeleton ratio. Myosin-II is a motor protein that is connected to the viscoelastic properties of the cytoskeleton. The cytoskeleton also has an influence on focal adhesions and adherens junctions [38,46].

Podosomes through the secretion of matrix metalloproteinases (MMPs) and invadopodia (actin-rich protrusions of the plasma membrane) degrade the ECM, thereby promoting invasion of other tissues. Protrusions of lamellipodia and filopodia promote cancer cell invasion, generating traction forces that are needed for mesenchymal-mode migration. Blebs are formed when a stimulus weakens the F-actin cortex, whose disruption is driven by the increase of hydrostatic pressure generated inside the cytoplasm. Bleb expansion occurs simultaneously with the contraction caused by bleb neck formation. In turn, bleb retraction occurs through F-actin assembly at the bleb cortex and due to Ras homologous A (RhoA), Rho-associated coiled-coil kinases (ROCK), and myosin, producing traction forces that help the cell to move forward. In some types of cancer, blebs are used to invade [47]. The blebs are retracted by actomyosin contraction and induce the appearance of traction forces that move the cells forward. De Nicola et al. described that membrane blebbing is associated with apoptotic cell death [47,48,49]. The aggressive cancer types do not respond to antitumor drug-induced apoptosis, which may be affected by actin cytoskeleton structures [50]. Stress fibers, lamellipodia, and filopodia are considered to promote the survival of cancer cells. Actomyosin contraction is essential for sensing the mechanical environments surrounding cells [50]. EMT promotes cell invasion and metastasis, since this process is associated with the decrease of E-cadherin and the increase of N-cadherin expression, given that the latter is weaker than E-cadherin and weaken cell–cell adhesions [50].

**Figure 3:**
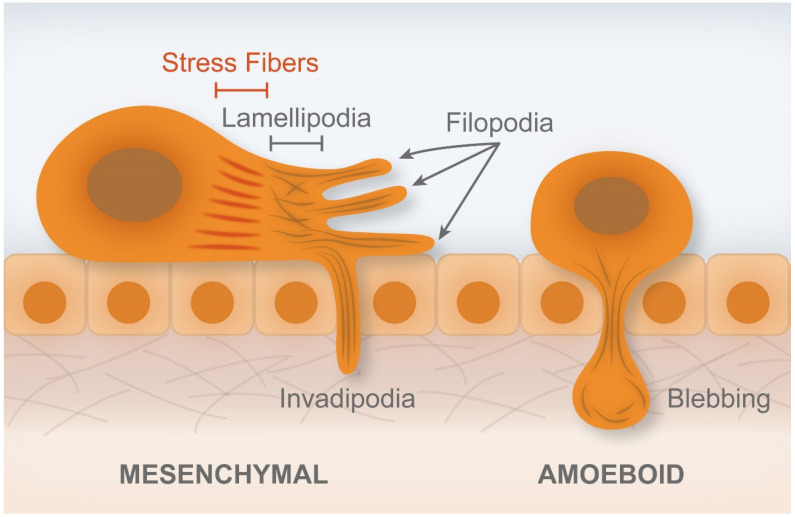
Cell migration though the epithelium and basal membrane (invadipodia) and through the reorganization of the cortex (blebbing). The cells are pushed in a certain direction due to the lamellopodia and filopodia forces produced at the leading edge. After receiving microenvironmental cues (such as growth factors, cell-cell contacts and extracellular matrix signals), invadopodia invade the neighboring tissues. Re-organization of cell cortex can induce blebbing, which facilitates amoeboid-like migration. Adapted from Biber et al. [53].

Pachenari et al. described the role of actin microfilaments and microtubules proportions in different grade I and grade IV CCCs, in which microtubules have an effective role in the reorganization of cytoskeleton in the transition from non-aggressive to malignant phenotypes [51]. According to them, when cells are forced to migrate through narrow pores in the ECM, high levels of actin-myosin contractile forces are transmitted to the nucleus, which could cause the DNA-repair factors to become mislocalized and ultimately increase the amount of DNA mutations in the cell [52]. This mechanically induced DNA damage may contribute to the cancer cell heterogeneity seen in tumor populations [37]. Biber et al. recently described actin regulators, such as diaphanous (Dia), and their role in CRC progression and metastasis [53]. The authors showed the abnormal expression of mammalian DIA-related formin-1 (mDia1) in colon carcinoma tissues in comparison with normal tissue [53]. Knockdown of mDia1 in CCC lines resulted in a compromised ability to perform adhesion, invasion, and migration in vitro. Furthermore, on mDia1-depleted cells, the ability of CCCs to invade adjacent tissues and extravasate from blood vessels was impaired. Interestingly, early cell adhesion defects observed in mDia1-depleted CCCs were due to microtubule dysfunction and not to actin dynamics under non-stimulatory conditions [54]. The injection of mDia1-knockdown cells resulted in significantly lower metastatic lesions in the lungs of animals [54]. Figure 3 shows the invasion of cells due to invadopodia, filopodia, and respective protrusions [53].

Kumar and Weaver reported that, during cancer progression, proteins of the cytoskeleton induced changes of mechanical properties in cancer cells regarding contraction, stretchability, deformability, and viscoelastic parameters in general [55]. Microtubules, microfilaments, and intermediate filaments are cell structures that regulate these processes [56]. A good biomarker of cell state could be cytoskeleton reorganization during cancer progression, providing crucial information for new developments in cancer diagnostics, preventive actions, new therapies, and better drug efficiency [57]. Studies of single-cell stiffness designed to investigate the contribution of cytoskeleton biopolymers (e.g., from microtubules and microfilaments) to the mechanical properties of cancer cells are fundamental [58]. Janmey et al. reported that the highest cell resistance to deformation was provided by F-actin up to a critical strain value [59]. This characteristic indicated that actin networks at the cell cortex enabled cell locomotion. They are also known to react quickly to external forces, having an important effect on the formation of leading-edge protrusions during cell motility [60]. In fact, microtubules are the second major constituent of the cytoskeleton acting with the other filaments, in the stabilization of cell structure in compression loading. In ovarian cancer, metastatic cells have shown less distinct F-actin structures, with thin stress fibers and less defined and disorganized microtubules [61].

#### 2.1.3. Cell Membrane

The cell membrane also influences cellular mechanotransduction, since it is composed of several proteins which sense the forces from the neighboring cells and from the ECM. They are the “bridges” sensing the forces from the microenvironment to the cytoskeleton, once the forces are transmitted from the mechanosensor membrane proteins, such as integrins. For example, tension is known to induce alterations in the cellular membrane, in the regulation of ion channels, playing a crucial role in cell locomotion, shape modification, and volume adjustment [46]. Ren et al. recently studied the effect of membrane tension, by AFM, through the use of the standard solid model applied to the cytoskeleton, which allowed for the quantification of the viscoelasticity through the force-curve approach as a function of time (time relaxation) [62]. The decrease in cell stiffness is associated with changes in the cell membrane surface. The authors showed that approximately 5% of the force exerted by the cell on the probe was from the cytoskeleton and the remaining force was coming from the cell membrane. Hence, it can be hypothesized that the reduction of the cancer cell stiffness can be due to the decrease of the cell membrane tension [62].

Integrins are transmembrane proteins that are connected to the ECM. Laminin-5γ2 (LN-5γ2) plays an important role in the tumor budding of CRC, due to the interaction between LN-5γ2 and integrin β1, which promotes it via the activation of FAK and yes associated protein-1 (YAP). Cucurbitacin B can block the interaction interface between LN-5γ2 and integrin β1, substantially inhibiting tumor budding [63]. Adherens junctions are known to be involved in sensing the mechanical microenvironments of cells, similarly to focal adhesions [64]. EMT has been described as a cooperation of a complex network and it includes factors classified in three groups: EMT inducers (extracellular cues), EMT core (transcription factors arranging the EMT program), and EMT effectors (effector molecules executing the EMT-related cellular transformation) [65]. The best characterized external inducers are the transforming growth factor-β (TGF-β) signaling, rat sarcoma viral (RAS), and the Wnt/β-catenin pathway [66]. The overexpression of genes encoding for proteins linked to a mesenchymal phenotype, such as vimentin, fibronectin, α-smooth muscle actin, and N-cadherin, and the down-regulation of epithelial markers, such as E-cadherin, claudins, and occludins, constitute the final effects of EMT regulators [67]. Bellovin et al. mentioned that studies in breast and colon cancers indicate that the cytosolic expression of p120 controls the invasive phenotype of E-cadherin negative cells [57]. Ieda et al. reported that red fluorescent protein (RFP) expression is driven by mesenchymal vimentin promoter. An inflammatory environment induced RFP expression in association with the EMT phenotype in CRC cells, demonstrating the distribution of RFP-positive CRC cells in rectal and metastatic tumors [68].

According to Boesch et al., the epithelial cell adhesion molecule (EpCAM) is present in the surface membrane of healthy epithelial cells [69]. EpCAM plays an important role in the EMT process. It is a transmembranous glycoprotein, consisting in an extracellular domain (EpEX) and an intracellular one (EpICD). It has been shown that the activation of the β-catenin/c-Myc pathway is induced by the shedding of EpICD to the nucleus, thus resulting in tumor proliferation. The overexpression of EpCAM is associated with cancer progression and a poor outcome [70]. In healthy tissues, the expression of EpCAM is limited to the basolateral membrane of epithelial cells and presents adhesions facilitated by cadherins, being absent in the tight junctions and desmosomes [71]. Winter et al. show that the shift of adhesions from strong to weak is caused by EpCAM modulation of cadherin-mediated contacts by antagonizing E-cadherin [72]. EpCAM can also interact with the cytoskeleton, binding to actin filaments. EpCAM is involved in the disruption of the interaction between actin filaments and α-catenin. Defects in cell-to-cell adhesion promote proliferation, migration, and differentiation, with EpCAM being a pro-metastatic molecule. EMT in tumor cells causes a downregulation of EpCAM, which is believed to induce motility and cell migration [73]. The overexpression of EpCAM is also associated to resistance to anticancer drugs in CRC [74]. EpICD can interact with α-actinin. The existence of cancer stem cells (CSC) (tumor cells that, through the EMT, adopt migratory potential) and EMT is a strong combination for metastatic CRC progression. CSCs detach from the primary tumor and intravasate into nearby vessels, to survive in circulation and extravasate to form metastasis in organs, such as the liver and lungs. It seems that CSCs are more efficient in metastasis formation since EMT cells first need to undergo the reverse process, i.e., mesenchymal-to-epithelial transition (MET), before engrafting. As a consequence, CRC progression is driven by CSC (EpCAM positive) and EMT (EpCAM negative) and also by the EpCAM expression [69].

#### 2.1.4. Stroma and ECM

The stroma is composed by an insoluble, often highly crosslinked, complex network of fibrous proteins (collagen, elastin, fibronectin, and laminin), glycoproteins, and proteoglycans (chondroitin, heparin, keratin sulfates, and hyaluronic acid) surrounding tissue cells [37]. When compared with cancer tissue, there is an increase of several proteins and cells, such as fibroblasts, immune cells, epithelial cells, ECM, and other proteins (e.g., fibronectin). Basically, the cancer niche is composed by the cancer cells in the middle, the basement membrane (BM) around, the stroma, and the ECM. If the BM membrane is disrupted, the cancer cells migrate through the stroma and ECM. Breaking the BM of the organ, cancer cells will intravasate into the blood or lymphatic node system.

Hence, the BM constitutes a physical barrier for avoiding the spread of primary tumor to adjacent tissues at the stage of carcinoma formation [75]. BM is composed of laminin and type IV collagen networks, produced by coordination between the epithelial cells and stromal fibroblasts [76]. It provides structural support to the epithelium, promotes cell adhesion, sustains cell polarity, and it is essential for tissue compartmentalization by separating the epithelium from the stroma [77]. Cancer cells perforate the BM using MMP-rich protrusions, called invadopodia, and stromal cells can also contribute to this process since they also produce matrix proteases [78,79,80]. CRC-specific matrix-associated molecules include intestinal receptors, proteases, mucins, and lectins, all contributing to the tissue stiffness [81].

**Figure 4:**
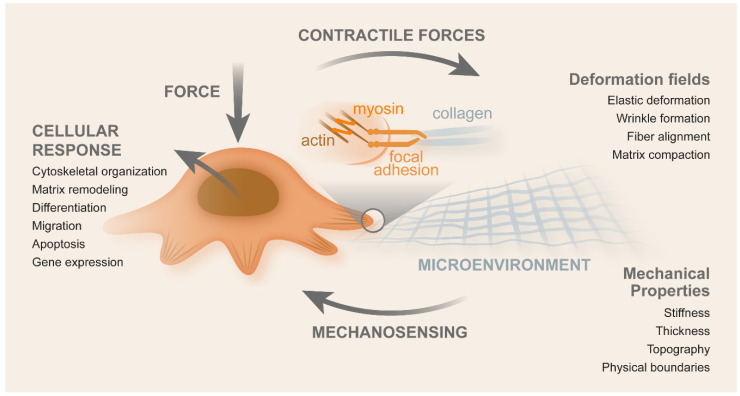
The feedback loop of force-deformation in cells and microenvironment. The cells sense the influence of the ECM microenvironment, such as stiffness, thickness, topography, and the physical boundaries with other cells (mechanosensing). These forces are transmitted to the interior of the cell through molecules that are bound to the cytoskeleton. The cytoskeleton transmits those forces to the nucleus, which will initiate the mechanics of cell response due to the activation of several gene expression pathways, that, in turn, will promote cytoskeleton organization, matrix remodeling, differentiation, migration, and apoptosis. The cytoskeleton organization and migration will produce the polymerization of F-actin filaments and the contraction of myosin motors, promoting cell motion, elastic deformation, and fiber alignment. Adapted from Mohammadi and McCulloch [84].

The ECM can be characterized by its topography, roughness, and stiffness. The latter can be defined as being the resistance that a material offers to the elastic deformation when a uniaxial stress is applied [82], while the elastic modulus of a material corresponds to the slope of its stress–strain curve in the elastic deformation region [83]. In response to applied force, cells will respond with cytoskeleton organization, ECM remodeling, gene expression, differentiation, migration, and apoptosis. Due to contractile forces, cells induce viscoelastic deformation, wrinkle formation, fiber alignment, and matrix compaction (Figure 4) [84].

The ECM provides both mechanical and biochemical signaling cues, thus being an indispensable and dynamic structure and directly affecting cellular behavior. Interruptions can occur either in the physical (e.g., porosity), biochemical (e.g., growth factor binding capacity), and/or biomechanical (e.g., stiffness) properties of the ECM. Some of these properties support and regulate cell differentiation, adhesion, survival, migration, and proliferation rates. During cancer progression, a deeper understanding of the ECM compositional changes is crucial to develop better and targeted treatments [85].

Mohammadi and Sahari used hydrogels to study how they affected the phenotype and gene expression of cancer cells [86]. Tang et al. indicated that increasing substrate stiffness promoted the metastatic phenotype in colon carcinoma cells [87]. CCCs were characterized by metastatic hallmarks, such as weak attachment to substrates and cells (after culturing on stiff substrates for seven days) [88]. This suggested that substrate stiffness may be a crucial parameter in the pathogenesis of cancer invasion. An increase in stiffness has been associated to the cell spreading area and size of focal adhesions [84], highlighting the importance of single-cell measurements to define the relationship between cell mechanics and the formation of cell adhesions. This process could be important to understand how cancer cells interact with ECM of varying stiffness values. In ECM stiffness, the elastic and inelastic behavior must be taken into account, which may modify mechano-sensation by cells [84]. A possible approach for therapeutic intervention seems to be preventing or reversing tumor stiffening since the stiffness of the tumor microenvironment contributes to tumor growth and metastasis [86].

Pinto et al. reported macrophages as additional key players in cancer progression, with the capacity to modulate cancer cell migration, invasion, and metastasis, with these immune cells being highly plastic, possibly adopting numerous and distinct polarization phenotypes, namely either pro- or anti-inflammatory [89,90]. Along with these cells, decellularized ECM has been described as having relevance in cancer progression. Human CRC and non-neoplastic mucosa were efficiently decellularized. Before repopulation with primary human monocytes, they were studied regarding their viscoelastic properties using rheometry. The tumor decellularized matrices presented higher stiffness than normal decellularized matrices [89]. They further showed that normal and tumor decellularized matrices constitute excellent scaffolds for recreating microenvironmental features, leading to a better understanding of mechanisms of disease and therapeutic resistance [89].

Several distinct studies also reported that cell properties depend on substrate stiffness [87,91,92,93,94]. CCCs modify their mechanical properties to resist intravasation, shear stress associated with circulation, and extravasation [95]. Mechanical cues can also play an important role in the early phases of metastases by adapting to the tumor microenvironment. The stiffness of ECM obtained through the crosslinking of collagen was achieved by modeling integrin’s expression and resulted in the induction of malignant behavior in mammalian epithelial cells [96]. One cause of tumor stiffness due to variations in the tumor microenvironment is the unbalance between ECM production and its degradation. According to Peddareddigari et al., in CRC, there is the production of MMP-1, -2, -3, -7, -9, and -13. When an increase in MMP-3 production occurred, CRC presented microsatellite instability (with changes in small portions of chromosomes) with a poor prognosis [66,81]. Meanwhile, when an increase of MMP-12 production was observed, there was a good prognostic of survival since this molecule had an inhibitory effect on angiogenesis [81].

In the process of strain stiffening the resistance of crosslinked ECM increases with increasing matrix deformation, through which the ECM undergoes cell-induced deformation. The rate and magnitude of matrix deformation determine the amount of plastic deformation. Slow rates of force-mediated collagen remodeling cause a stable alignment of its fibers, which is related to poor outcomes [97]. Proteolytic cleavage by MMP-14 can indirectly counteract the effect of crosslinking by decreasing matrix stiffness [98]. It is likely that cycles of proteolytic degradation, new synthesis, and new crosslinking, enable permanent matrix remodeling which is mainly elastic over long-time scales [86].

Nebuloni et al. investigated the biochemical and physical diversity of ECM in healthy, perilesional, and CRC samples. The authors observed that ECM from perilesional and CRC samples withstood the proliferation and invasion of tumor cells. From healthy ECM to perilesional and CRC, a progressive linearization and organization of fibrils was observed, associated with an increased collagen crosslinking and consequent increased stiffness. In perilesional ECM, these modifications corresponded to an increase in vascularization, whereas in neoplastic ECM they were associated with the altered modulation of some proteins [99]. This study has shown that the increase in stiffness and crosslinking of perilesional ECM favors a suitable environment for CRC invasion associated to the vascularization process. The stiffness increase of colon tissues may be used as a marker of desmoplastic tissue predisposing to invasion, which can be used as a new potential application for follow-up of adenoma with invasive potential [99].

In 2D models, it is easier to study these parameters but not angiogenesis (new vessels formation from pre-existing ones), since it cannot be reproduced in a 2D model. The angiogenesis process is an essential step when a tissue mass transitions from a benign state to a malignant state, meaning that if the oxygen is missing the tumor becomes hypoxic.

Three-dimensional (3D) cell culture models are an emerging powerful approach for studying ECM mechanics and cellular responses, although they still lack the cell and matrix complexity of natural tissues. Reidy et al. described the evolution of 3D models used in CRC studies, as well as their advantages and disadvantages. They showed their utility to predict the response to therapies and resistance. It was shown that an increase in stiffness promoted CRC progression and difficulties in drug delivery, thereby promoting resistance [100]. Moreover, 3D studies are fruitful since they provide a more similar environment to the native structure (such as 3D geometry, porosity, ECM binding site, ECM heterogeneity, and gradients of biochemical factors, among others), although they recognize that there are several challenges remain to be overcome, namely the difficulties in controlling the environment and in visualizing cell–cell and cell–ECM interactions, as well as in quantifying results without specific imaging technology [101]. Lysyl oxidase (LOX) has already been used to increase the collagen matrix stiffness in order to promote relevant physiological conditions. Hydrogels are known to have poor mechanical properties. However, when methacrylamide and methacrylate groups are incorporated, stiffness increases significantly, mimicking the CRC microenvironment [100]. Lysyl oxidase (LOX) has already been used to increase the collagen matrix stiffness in order to promote relevant physiological conditions. Hydrogels are known to have poor mechanical properties. However, when methacrylamide and methacrylate groups are incorporated, stiffness increases significantly, mimicking the CRC [100].

### 2.2. Mechanics of CRC Cells

Cells respond to internal force generation, as well as to the change of mechanical properties of their surroundings. Internal forces contribute to cell motility, stiffness, focal adhesion, and shape changes. Cells respond to external stimuli transmitted by the ECM (through stiffness) by changing their Young’s moduli, maturation, and focal adhesion number. The responses mentioned previously were observed through a range of mechanisms, namely integrin activation and reinforcement as well as the activation of putative force sensors, stretch-activated channels, protein unfolding, and activation of signaling pathways [102,103]. Cell surface roughness, shape, actin organization, stiffness, and adhesion are modulated by CRC cells to better accomplish their specific tasks in cancer growth and invasion [104].

In cell biophysics, cell morphology, shape, and roughness can also provide significant information since these parameters may affect the mechanical properties in disease. Boccaccio et al. used nanoindentation by atomic force microscopy (AFM) to study the effect of surface roughness and cell shape in mechanical properties variation of SW480 and SW620 cell lines on colon cancer and lymph-node-derived metastasis, respectively [105]. The authors found differences relative to roughness properties for the SW480 (round, R shape and elongated, E shape), showing that rounded cells (SW480R and SW620) possessed comparable elastic parameters, which were different from those of elongated SW480E cells. This conclusion allowed them to formulate the hypothesis that the properties of rounded cells are intrinsically different from those of the elongated ones [105]. This could also contribute to the hypothesis that the role of subcellular components is different from one cell to another. Palmieri et al. also observed that SW480 populations have specific functions when proliferating in the primary site (SW480R) and metastasis (SW480E), being reflected in cell shape and mechanical properties. The R-type cells showed a decrease in stiffness due to the actin anisotropy, as opposed to E-type cells [104]. The SW620 cell type, with its round shape, took advantage of its high adhesion capacity (shown between cells and AFM tip) to metastasize, by better adhering to the blood vessel walls. Regarding shape and topography, studies developed by Ball et al. in osteoblast-like cells showed the shape factor changes with respect to substrate morphology where the cells were seeded [106]. In terms of the quantification of cell elongation, a shape factor of 1 corresponds to circular cells, and a factor of 0 corresponds to elongated cells.

Tang et al. studied three different human CCC lines (HCT-8, HCT-116, and HT-29), all of them having a low metastatic potential and an epithelial elongated phenotype (E-type) [93]. When these cells were seeded on a stiff substrate, they adhered, spread, and proliferated, forming a monolayer with E-cadherin-mediated junctions. A very rare, small number of round-shaped cells (R-type) was observed on top of these monolayers. However, if the HCT-8, HCT-116, and HT-29 cells were seeded on a soft substrate, 50–70% of these E-type cells transited to R-type. This confirmed that the stiffness of substrate has implications in cancer cell metastization [93].

**Figure 5:**
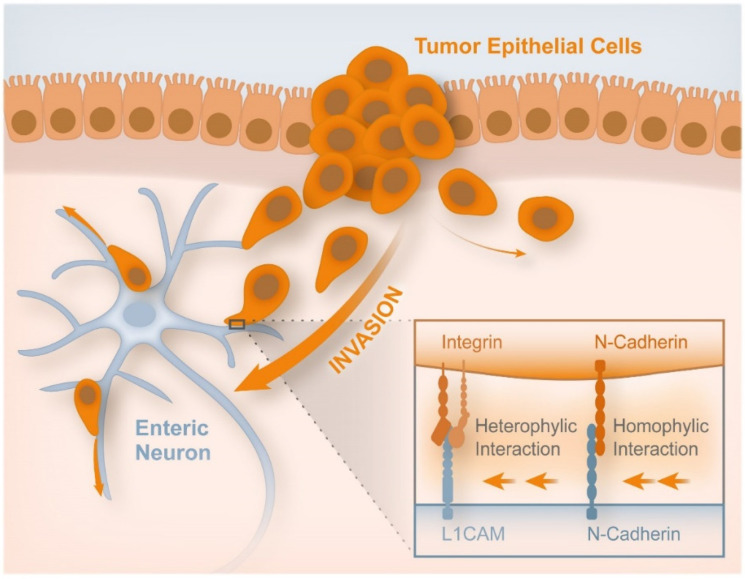
Cells involved in the adhesion and migration of CRC cells through the local enteric neurons network. It is speculated that tumor epithelial cells migration is performed along the neuronal fibers, through the heterophilic bond of L1CAM—integrin and suggesting the existence of homophilic bond of N-cadherin. These physical process guides the migration of CRC cells. Adapted from Duchalais et al. [111].

Tang et al. showed that when cultured on soft substrates (21 KPa), human colon carcinoma cell line HCT-8 changed from low to high metastatic state and thus lost mechano-sensitivity as they underwent E-to-R transition [93,107]. Cell spread area, proliferation, and migration of HCT-8 R cells were not sensitive to substrate stiffness changes in contrast to E cells. R cells acquired autonomy and anchorage independence, being more invasive than E cells [108]. Lammermann et al. recently described the different amoeboid phenotype based on the force relationship of the three major forces in cell migration, i.e., adhesion, contraction, and polymer network expansion [109]. Panzetta et al. showed the inherent amoeboid appearance of the S174T colon carcinoma cell line [110]. Duchalais et al. demonstrated, by AFM, that tumor epithelial cells (TEC) have stronger adhesion forces to enteric nervous structures than to mesenchymal cells [111]. TEC adhesion to non-myelinated intrinsic enteric nervous system (ENS) involves direct interactions with enteric neurons. The removal of enteric neurons from ENS cultures resulted in a significant decrease in tumor cell adhesion. ENS significantly promoted TECs migration when compared with migration through mesenchymal cells. TECs migration followed faithfully the trajectory of the ENS structures. The enteric neuron used the neural cell adhesion molecule L1 (L1CAM) and N-cadherin to bind to CRC. The authors hypothesized that CRC cells bind to the L1CAM of ENS using an integrin since the binding of L1CAM to α5β1-integrin was reported in other organs. The authors also mentioned the presence of N-cadherin expression in both TECs and ENS, suggesting homophilic binding. A possible local path for CRC spreading may be the dense network of local enteric neurons in the CRC microenvironment [111]. Figure 5 shows the cells involved in the adhesion and migration of CRC cells through the local enteric neurons network [111].

### 2.3. Techniques to Characterize Mechanotransduction

For mechanobiology and mechanotransduction studies, several techniques can be used, such as AFM, microindentation, shear rheometry, shear wave and magnetic resonance elastography, micropipette aspiration, optical stretching, and magnetic twisting cytometry, which have been developed to evaluate the mechanical properties of cells and tissues [82]. Table 1 presents different techniques used in mechanotransduction studies, including a brief summary on each of them. The characterization techniques mentioned below allow the quantification of biophysical parameters, from cells up to solid and irregular biologic samples, in opposition to the histopathology and immunohistochemistry assays, which are the standard methods still in use but result in the loss of mechanical properties after tissue fixation.

Recently, Deptula et al. used AFM and shear rheometry as potential techniques for cancer diagnosis by studying the mechanical properties of human colon cancer fresh tissues, as possible mechano-markers in the oncological clinical field for predicting the stage of disease [112].

AFM was also used by Cross et al. to study the morphological changes and nanomechanics of adenocarcinoma cells (from breast and pancreas), lung carcinoma cells, metastatic cells found in the body pleural fluid, and compared them with the mesothelial cells (cells that line the body cavity). This work showed that that the stiffness of carcinoma cells was lower than that of mesothelial cells, in spite of them having similar shapes. This work also showed that the nanomechanics analysis fitted well with the results of the immunohistochemistry used for cancer detection, demonstrating that mechanical quantification can be a biomarker in cells removed from body fluids and not only for solid tumors [125].

### 2.4. Biophysical Cues vs. Biochemical Signaling

Thanki et al. referred the classification of CRC according to the consensus molecular subtypes (CMS): CMS1 is immunogenic and hypermutated, CMS2 has the highest overall survival and is activated by the Wnt/β-catenin pathway, CMS3 presents a metabolic phenotype, while CMS4 cancers present a stromal gene signature and the worst survival [126]. Each CMS is characterized by the activation of different signal transduction pathways. The CMS classification provides information to clinicians about prognosis, therapeutic response, and new insights for the development of therapeutic strategies [126]. Singh et al. also referred two different molecular classifications of CRC through the candidate cancer gene (CAN) [2].

In the case of CRC, Pandurangan et al. and Smit et al. mentioned that the main signaling pathways affected in CRC epithelial cells are: the APC, KRAS, transforming growth factor-β (TGF-β/Sma genes and the Drosophila Mad (SMAD4), protein 53 (P53), Wingless-related integration site (Wnt)/β-catenin, YAP/Transcriptional co-activator with PDZ-binding motif (TAZ) hippo signaling, Rho-family, Tumor necrosis factor-α (TNF-α), and phosphoinositide 3-kinase (PI3k) pathways, which are known by the adenoma-carcinoma sequence (ACS) [3,127]. It seems that there is an optimal balance between mechanical and genetic cues and that plays a key role in the genesis and development of malignancies [56,128,129]. As a consequence, a balance between the biomechanical and biochemical properties of cellular components and ECM alters cellular behavior, stimulating biochemical pathways which will be drivers of cell proliferation, migration, invasion, survival, and metastization [130]. Some biophysical stimuli activate more than one biochemical pathway response. Some activation pathways induce the activation of other sequence pathways, which means that in CRC there is a co-existence of several pathways, almost simultaneously.

The epithelial proliferation is associated to the mutation of APC gene, a key tumor suppressor gene. Loss of APC function leads to colorectal tumors [95]. Whitehead et al. applied mechanical strain (deformation, in z-direction, 20 min with a load of 800 Pa) on colon explants from healthy and APC-deficient mice. Tissues showed shape changes at the cellular level, which were proved to be to be coincident with the expression of two oncogenes (Myc and Twist1) in tissue explants with APC deficiency, but not in the wild-type colon explants. This can be explained by the fact that with the mechanical deformation, the APC deficient tissue was associated to an increased number of β-catenin positive nuclei per crypt, but this fact was not detected in wild-type mice colon epithelium [131]. This mechanical strain can be associated to the intestinal transit and, when APC is down regulated, can favor the presence of polyps and tumor growth [132]. It is known that APC can also be overexpressed and cause CRC, which means that APC has a dual role in the CRC cancer. It is crucial to know more about these two opposite mechanisms to develop suitable therapeutic strategies [133,134]. The expression of APC promotes cell asymmetry due to the longer cellular protrusion formation [135].

Zanconato et al. also corroborated that most CRC cases carried mutations leading to the Wnt signaling pathway, among which were mutations inactivating the APC tumor suppressor [36]. Wnt pathway was mentioned by Ciasca et al., reporting the mechanical cues that may contribute to early phases of tumor initiation, since that regulator is one of the most important in cell proliferation [136]. The same authors described well the two stages of the Wnt pathway (on and off) leading to cell proliferation [95]. In a study with 224 CRC cases, in 94% of them, a mutation in one or more members of the Wnt signaling pathway was detected [137]. Lee et al. corroborated that the Wnt/β-catenin cascade plays the dominant role in controlling the fate of epithelial cells in the intestine and colon, as well as a mutation in the APC. Additional mutations, such as the human gene that encodes a protein called B-Raf (BRAF) protein pathway, resulted in the growth of a small adenoma with clinically significant size (>1 cm) [13,137]. Additional mutations in p53 drive the malignant transformation to adenocarcinoma. Stages of CRC development and the extent of disease were associated with the activations of cyclooxygenase-2 (Cox-2), EGF, and VEGF [13].

The p53 mutation was found in ca. 60% of CRCs [138]. In patients with CRC, the apoptosis of p53 proteins is downregulated [139]. This mutation is sensitive to ECM stiffness. In integrin signaling pathways, the downregulation of molecules suppressed cancer progression due to p53 wild-type. Ebata et al., on the contrary, documented that the molecules upregulated by p53 mutants were α5, 𝛽1, and 𝛽4 integrins and fibronectin. It has been revealed that, by regulating the expression of E- and N-cadherins, p53 prevents EMT [50,140].

In CRC, KRAS is the most frequent isoform mutation, accounting for about 20% of all human cancers, while neuroblastoma rat sarcoma (NRAS) and Harvey rat sarcoma (HRAS) mutations are found in about 8% and 3% of cancers, respectively [66]. The involvement of RAS in cellular plasticity was demonstrated by morphological and molecular correlations. Invasive and migration properties of malignant cells influence cellular plasticity. The initiation of EMT in CRC involves RAS signaling, leading to tumor spreading. According to the adenoma-to-carcinoma sequence, KRAS and p53 genes are regularly mutated. Mutations of KRAS were able to promote the development of CRC, while NRAS mutations were not, as investigated in an APC-deficient mouse model. The RAS function has been well described by Maffeis et al. [66].

Hippo signaling can be ON or OFF [50,141]. If Hippo are ON, this is corresponding to YAP/TAZ being inactivated. If Hippo signaling are OFF, this corresponds to YAP/TAZ being activated [142]. YAP/TAZ is mis-expressed in several solid cancers such as CRC. High expression of nuclear YAP is related with tumor progression and survival decrease. However, a dual role of YAP has been recognized both as an oncogenic and as a tumor suppressor in differential phases of CRC progression [143]. Ma et al. reported that hypoxia-inducible factor (HIF) was present at the intestine and HIF-2α was essential for CRC growth and progression. The authors found that HIF-2α activated YAP1 was crucial for cell growth during hypoxic stress in colon-derived cell lines [144]. However, Ou et al. described the dual role of YAP, i.e., both the oncogenic and tumor suppressor. To understand these two mechanisms is important for the development of CRC therapeutic strategies [143]. At cell–ECM adhesion sites, where the cell geometry and matrix stiffness are noticed, the YAP plays an important role in signaling activity [145]. In animals, the Hippo signaling pathway is considered the main transcriptional effector, being identified as a key mechano-transducer, performed by nuclear tips of mechanical stimuli [146]. Cellular proliferation and inhibition of apoptotic signals, loss of contact inhibition and tumor growth, and increment in cell size and organ growth were associated with YAP/TAZ accumulation and its activation in the nucleus [146]. Elosegui-Artola et al. highlighted that ECM–nuclear mechanical coupling translocates YAP in response to substrate rigidity [147]. The authors explained that, using soft substrates, the forces applied directly to the nucleus induced YAP nuclear translocation while decreasing mechanical restrictions to molecular transport from nuclear pores. When cells were exposed to a stiff environment, they established mechanical connections through the forces exerted by focal adhesions from the cytoskeleton to the nucleus [147].

TNF-α has been described as another signaling pathway to be taken into account in CRC. Liu et al. used AFM to study the mechanical properties of EMT in HCT116 human CCC lines, in the presence and absence of TNF-α treatment, since chronic inflammation is considered the seventh hallmark of cancer [148]. TNF-α is widely recognized as among the main mediators of cancer-related inflammation, mediating all steps of tumorigenesis, such as proliferation, invasion, angiogenesis, and metastasis formation, by accelerating tumor invasion and metastasis through EMT. TNF-α induced morphological changes consistent with EMT in HCT116 cells, since cells with no TNF-α treatment adhered to each other, while after treatment, cells exhibited morphological changes and intercellular spaces [148]. These findings suggest that it is crucial to investigate medical substances to avoid morphological changes in CCCs consistent with the EMT, thus avoiding the existence of (or at least part of) conditions to metastization. It is not well known which biophycal cues can activate this pathway.

Zessner-Spitzenberg et al. investigated the role of TGF-β in shifting the epithelial cancer cells towards a pro-migratory phenotype via stromal signaling and, consequently, invasiveness [138]. A poor prognosis subset of CRC was identified in some studies, which was characterized by high stromal activity and elevated levels of TGF-β [149]. This protein has been recognized as a main driver of CRC metastasis [138].

Tsuji et al. reported differences in terms of microvessel growth in the tumor tissues, in relation to amounts, diameter, and spatial direction in carcinomas with metastasis and without metastasis [150]. The authors also found significant differences related to the presence of VEGF and reported a higher VEGF-positive cell count in tumor tissues when compared to normal tissues. With tumor progression, microvessel diameter showed a significant increase and microvessels counts a decrease, which can be partially explained by the VEGF expression. In the first step of metastasis, the microvessel diameter seems to be the predominant parameter responsible for intravasation cell cancer [150].

Ebata et al. reported the importance of Rho family activation in cancer metastasis [50]. Rho GTPases, such as Rho, Ras-related C3 botulinum toxin substance 1 (Rac), and cell division control protein 42 homolog (Cdc42) regulate the activation of actin regulators, contributing to the formation of filopodia, lamellipodia, and stress fibers, respectively [141]. The formation of actin-mediated structures, such as lamellipodia, filopodia, podosomes, and invadopodia, is associated with the invasion of cancer cells [140,141]. Rho signaling is related to the ECM stiffness and the ECM proteins have an important role in the activation of several biochemical pathways. Gagné et al. recently demonstrated that integrin-linked kinase (ILK) specifically induced the initiation of fibronectin, fibrillogenesis, during cell spreading, promoting the Rho/ROCK-dependent cell contractility and maturation of the integrin-actin structures [151]. According to them, fibrillogenesis and its effect in Rho signaling, cell contractility, and spreading did not depend on ILK in epithelial crypt cells in the human intestine [151]. Fibronectin interacts with an ECM glycoprotein named tenascin-C, participating in cell migration and organogenesis. Tenascin-C is crucial in the activity of growth factors, proteases, and protease inhibitors during cell spreading. This protein also induces Rho A/ROCK-dependent cell contractility and the integrin–actin axis structures [152]. Fibrillogenesis, cell contractility, and spreading are independent of integrin and kinase in epithelial cells crypt in the human intestine [151]. Krndija et al. mentioned that the Rho family also mediated the contractibility, number, and size of adhesion sites, as well as the increase of stress fibers [153]. The generation and response forces were identified as key elements in cell migration. The authors reported that the expression of receptor-type tyrosine-protein phosphatase alpha (RPTPα) in human SW480 CCC line was related to cell contractibility, a process in which there is an increase of that protein, thus increasing the number and size of adhesion sites and stress fibers (bundles of F-actin and myosin II held together by crosslinking proteins, thus ensuring the cytoskeletal contractility) [154]. RPTPα influenced cell spreading on low-rigidity surfaces, suggesting that force-responsive proteins can influence cancer cell behavior and identify potential novel targets for cancer therapy [153].

PI3k signaling is related to cell growth, differentiation, cell motility, intercellular trafficking, and cellular survival. This signaling pathway is not activated directly through a specific biophysical stimulus. It is activated as a consequence of other activations of biochemical pathways [95,155].

Table 2 shows some examples of putative genes which are influenced by biophysical cues, inducing the activation of biochemical pathways. These biochemical pathways, in turn, will influence the cell behavior. Prognoses were also able to take into consideration the CMS classification. Some possible therapies were indicated. The signaling pathways that have a dual role (APC, YAP/TAZ, and Wnt) in CRC are highlighted with different colors (blue corresponding to under expression or loss and red corresponds to overexpression of the pathway).

### 2.5. Role of Cancer-Associated Fibroblasts (CAFs)

Fibroblasts produce the ECM and the collagen fibers. Fibrosis is defined as a normal reaction in the wound healing process, in which there is an excessive accumulation of ECM proteins. In cancer, it is expected that condensed collagen works as a barrier to avoid the spread of the cancer cells [165]. Bremnes et al. described how the fibroblasts are activated to CAFs in tumor disease [152]. CAFs, being the major constituent of the tumor stroma, play an important role in cancer progression [152]. CAFs activity is regulated by various cancer-associated growth factors, connected to the metabolism, proliferation, and metastization of cancer cells [166,167]. Shin et al. evaluated cancer invasiveness and the reactions of abnormal cells and their relationship with CAFs in long-term in CRC patients [165]. The authors concluded that the percentage of mature CAFs in the intra-tumoral stroma and the invasive front were 57.6% and 60.3%, respectively. The mature CAFs in the invasive front presented a significantly higher amount of epidermal growth factor receptor (EGFR), when compared with the immature CAFs. Hanahan et al. described that the increase in the number of mature fibroblasts implicated the increase of lymphatic invasion in the intra-tumoral stroma. With the progression of the tumor, the surrounding microenvironment became enriched in CAFs, cells from the immune system, and ECM [168]. Gaggioli et al. studied cell invasion in an animal model and showed that CAFs were responsible for cancer cell invasion through the type I collagen/matrigel hydrogels [169]. Cancer cells sense the physical forces exerted by CAFs, through the heterotypical cell–cell interactions, which stimulated their invasion [170]. Glentis et al. explained that in carcinoma in situ, the BM segregated tumor cells from the stroma [171]. For tumor dissemination to the adjacent tissues, this barrier must be broken, which may be achieved by proteolysis. The authors have shown that CAFs promoted cancer cell invasion through the BM, independently of the activity of MMPs. The BM is also affected by CAFs, since BM is pulled, expanded, and softened by them. The formation of gaps contributes to cancer cell migration. These gaps are increased by the BM alteration, since CAFs modify the organization and the physical properties of the BM, allowing cancer invasion [77].

Belli et al. reported that the ECM stiffness of primary tumors may be affected by CAFs since it improves cancer cell invasion, inducing EMT, thus supporting the spreading of metastasis [77]. CAFs also express intercellular adhesion molecule 1 (ICAM1), with its functions of regulating immune response, programming cell death protein ligand (PDL)1 and PDL2, and mediating immunosuppressive functions [77]. Mierke recently presented a correlation between cells and their microenvironment, including CAFs and type I collagen fiber scaffolds [172]. The synthesis and deposition of collagen and released LOX and transglutaminase-2 exosome are influenced by CAFs, increasing the collagen stiffness [172]. Peng el al. recently reported CAFs resulting from the activation of fibroblasts, which were activated by integrin ανβ6 on CCCs [173]. Normal fibroblasts showed less parallel organization of fibronectin when compared with CAF-derived matrices [173]. Platelet-derived growth factor receptor-alpha (PDGFRα) and myosin-II-driven traction forces transduced by integrin α5β1 contribute to CAFs matrix organization [174,175].

**Figure 6:**
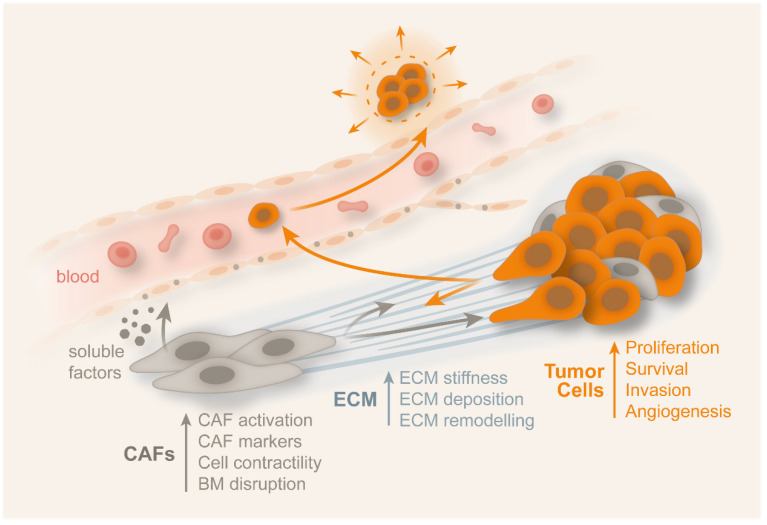
The stiffening and angiogenesis resulting from the remodeling of the ECM, is associated with the presence of cancer-associated fibroblasts (CAFs). Cancer cells at the leading edge of the tumor will assume epithelial-to-mesenchymal transition (EMT) and travel along collagen and fibronectin fibrils towards the vasculature. Due to CAFs, fibrils and collagenase are aligned at the edge of the tumor to promote invasion. The cell cytoskeleton will reorganize due to the shear forces sensed, eventually promoting adherence to the blood vessel wall. CAFs also contribute the disruption of the basement membrane. Adherent cells that survive will extravasate to a distant tissue. Cancer cells will not undertake mesenchymal-to-epithelial transition (MET), if the tissue is considerably stiff, and therefore the cells become dormant. MET will occur only if the elastic modulus is low and a secondary tumor will then be formed. With tumor progression, the matrix stiffness will increase. Adapted from Libring et al. [37] and Jang et al. [175].

Attieh et al. also referred that the CAFs, being the most abundant cells in the tumor stroma, have the capacity to contract the matrix and induce invasion [176]. The authors reported that CAFs contributed to fibronectin assembly and induced invasion through integrin αvβ3. If there was no fibronectin present, the contractibility of the matrix induced by CAFs was maintained but the ability to induce invasion was eliminated, meaning that CAFs retained the capacity to induce invasion in a fibronectin-dependent manner. CAFs produce a rich fibronectin ECM, with anisotropic orientation, which promotes cancer cell migration in certain directions [174]. New hallmarks of CAFs promoting tumor invasion can be defined in relation to the fibronectin assembly and integrin αvβ3 expression [176]. Periostin is a ligand for integrins αvβ3 and αvβ5, also being the CAF-released matricellular protein that induces angiogenesis. In several cancers, such as oral, ovarian, breast, and colon, periostin is believed to induce an improvement of cell survival growth, proliferation, migration, invasion, and angiogenesis, since the previous two integrins facilitate signaling pathways [175,177]. Periostatin also promotes promoting cell adhesion and migration. On colon cancer cells, only integrin αvβ3 interacts with periostin, facilitating metastatic growth and angiogenesis [52,175,178]. Staudacher et al. reported that, in CRC patients, the worse prognosis can be associated to the combination of activin-A and TGF-β expression. TGF-β increases the epithelial and stromal activin-A secretion, with migration being dependent on it [179]. Metastasis is also promoted by the upregulation of MMPs by activin-A. MMP-7 induction by activin-A is a requirement for dissolving BM and other ECM components to allow the metastatic spread [180]. According to Storm et al., CAFs may exert a significant force on the ECM which is generated by ROCK-dependent actomyosin, through integrin-mediated adhesions [181]. Calvo et al. reported that the stiffness of the ECM was caused by the tension forces due to actomyosin contractility, increasing the collagen tension at the tumor microenvironment [182]. Madsen et al. reported that the contractility of CAFs was reduced under hypoxic conditions [183]. Zhou et al. mentioned that tumor budding, being the existence of a single cell or clusters of up to five cancers cells, is an independent prognosis factor for CRC [184]. This process is still not clear and the drugs that could inhibit this process are still in a very early research stage [165,184]. Tumor budding was visualized in both mature and immature stroma samples, occurring frequently in infiltrating type tumors. EGFR overexpression, maturation of CAFs, and infiltrating tumor growth configurations conducting to potential tumor were associated to well-connected islands (Figure 6) [37,175].

### 2.6. Impact of Shear Stress on Metastization

To survive the mechanical stress associated with intravasation, circulation, and extravasation, the metastatic cells modify their morphology and mechanical properties, such as adhesion and stiffness. These metastatic cells result from the physical stimuli involved in the differentiation of non-invasive cells [95]. The surface of cells senses mechanical forces generated by fluid shear stress in the adhesion points through integrins 1 and 3, being decoded in changes of gene expression. Endothelial cells were used in most studies [95]. In CRC, progression of cell cycle is regulated by mechanical forces due to fluid shear flow [185]. Since cancers of intestinal tract grow into the gut lumen, they are wide-open to different degrees of fluid shear stress [186]. In CRC, it is controlled by mechanical forces which are promoted by fluid shear flow. A fluid flow of 0.4–3.5 × 10^−4^ N/cm^2^ is the normal range found in the intestinal epithelium during peristalsis of the gut. Avvisato et al. exposed SW480 cells to a flow shear stress of 1.5 × 10^−4^ N/cm^2^ and found that the expression of β-catenin decreased by shear stress, inhibiting the Wnt-pathway activity [185]. However, the expression of other genes, namely those responsible for laminin, were increased by shear stress [185]. After being exposed to a shear stress of 2.5 × 10^−4^ N/cm^2^, the SW480 cell line (which does not express E-cadherin) showed a decrease in β-catenin signaling, in which the α6β4 integrin functions as a mechanosensor for fluid stress modulation. The authors also found that the cell cycle on SW480 cell line was blocked in G1 by the fluid shear stress [185]. The importance of the actin and microtubules in cancer cell adhesion was reported by Suresh et al. [56]. The stable adhesion of cancer cells to the walls of small blood vessels influenced metastatic formation. Focal adhesions are involved in the cell signaling modulated by cyto-disruption, during in vitro flow conditions. These data led to hypothesize that changes in the cell stiffness and voracity of the cell adhesion molecules (due to disruption of the cytoskeleton) were very important for adhesion interactions in vivo [56].

Delon et al. recently studied the influence of a fluid shear stress using 0–9.93 × 10^−5^ N/cm^2^ on Caco-2 cell line monolayers with a microfluidic device [187]. Caco-2 cells experienced significant phenotypical and functional changes when exposed to fluid shear stress, when compared with cells studied in static conditions. The authors mentioned that fluid shear stress exposure altered the mucus production, tight junctions expression, cytoskeleton organization, microvilli formation, activity of mitochondria, and cytochrome P450 expression [187]. They highlighted that, while a minimum fluid shear stress was a requirement to promote the formation of tight junctions, the cell–cell tight junctions as well as the monolayer barrier functions decreased, due to the application of high mechanical forces on those monolayers [187].

## 3. Mechanobiology in CRC Therapeutics

Conventional therapies, such as chemotherapy and radiotherapy, have been developed based on biological and biochemical knowledge of cancer cells. However, physical properties have emerged as playing an important role to fight cancer. These conventional treatments have the disadvantages of affecting not only tumor cells, but inducing undesirable collateral effects in healthy cells and tissues. Cell membranes and the cytoskeleton of cancer cells are targeted by chemotherapeutic agents, inducing cytotoxicity and altering the adherence process. The effects of chemotherapy usually rely on altered cell mechanics and may lead to vascular complications. The causes of those complications are related to mechanical obstructions of blood vessels from the main organs. The obstruction is caused by the increase of circulating leukemia cells, as well as the increase of cyto-adherence and stiffness [56]. The only advantage of conventional treatments is that they show fast results (independently if they were efficient or not) and, if they are not efficient, clinicians may quickly change the drug (or combinations of drugs).

The biophysical effects (elastic changes) of chemotherapy were studied on acute lymphoblastic leukemia and acute myeloid leukemia cells (removed from peripheral blood or bone marrow of 8–78-year-old patients) [188]. Both cell types were treated with dexamethasone and daunorubicin. Nanoindentation assays by AFM showed that leukemia cells exhibited doubled stiffness values during cell death [188]. Cells taken from the patients but not exposed to chemotherapeutic drugs did not show significant changes in their elastic moduli. The stiffness increase seems to result from the reorganization of the actin microfilaments, due to the polymerization and depolymerization, during apoptosis [189,190].

Immunotherapy has the advantage of reinforcing the immunological system based on antibodies, vaccines, and adoptive therapies. The disadvantage is that results are not so quick as those obtained with chemotherapy and/or radiotherapy. Some cancers do not respond to immunotherapy [126]. The combination of immunology with biophysical studies has led to mechanoimmunology [191]. Chimeric antigen receptor-T cell (CAR-T) constitutes a promising method to recruit T-cells to fight cancer cells. The CAR-T is separated in the CAR-T intracellular domain and its plasma membrane, due to mechanical forces produced by membrane motions, which activate signaling processes. The CAR-T has been mentioned to be helpful when joined to hematology malignancies. This molecule is also interesting to be used in solid tumors, such as CRC. The objective is to promote the binding of T-cells to different antigens of the CRC cells [192]. AFM could be a suitable methodology to study the specific bindings of CAR-T cells and the tumor molecules. AFM single-cell force spectroscopy can be used to study the binding of the CAR-T and eventual antigens that could exist in CRC cells. Kristi et al. used AFM to characterize the molecular interaction forces between the immune cells system and the antigen-presenting cell [191]. Lysophosphatidic acid (LPA) is a ligand for cell surface receptors. CRC cells demonstrate an aberrant expression of LPA receptors, contributing to tumorigenesis through the increase of inflammatory responses [193]. High levels of LPA2 are expressed by human CRC tissues compared with corresponding healthy colonic tissues. In adenocarcinomas of the colon, the LPA1 expression is lower [194]. With the LPA receptors being very important in colon cancer, they should be one of the targets in terms of the development of new therapies, i.e., new antibodies that could block the LPA binding receptor or new therapeutics that stop the gene pathway responsible for the LPA expression. AFM is a powerful tool, since the probe can be functionalized with molecules such as antibodies to study the specific target receptors.

Cortes et al. using AFM nanoindentation (through measurement of elasticity), found that when a specific drug was used to treat pancreatic ductal adenocarcinoma, cell stiffness was reduced, thereby suppressing cell invasion [195].

CTCs provide information about physical (size, density) or biological (tumor markers) parameters found in the patient’s blood [196,197,198].

The SW480 cell line treated with fullerenol was studied by Liu et al. using AFM to demonstrate the morphological and mechanical changes [199,200]. The Young’s modulus was used together with the adhesion force to infer the resistance of cells to the administered drug. The multinuclear cells, treated and untreated with two different concentrations of the drug, showed similar characteristics regarding their height, length, width, and roughness. For mononuclear cells, the distribution of those parameters was significantly different. The authors observed that multinuclear cells are more resistant to the fullerenol when compared with the mononuclear ones [201].

Pachenari et al. studied the effects of albendazole, a microtubule-targeted drug, on CCCs [51]. The authors reported the difference in grade I and grade IV CCCs, in terms of the proportion of microtubules and actin filaments. Micropipette aspiration was used to assess the viscoelastic behavior of grade I and IV of SW48 CCC line (more aggressive) compared to HT29 CCC line (less aggressive). Using relative fluorescence revealed that the ratio of actin filaments to microtubules in SW48 cells was equal to 1.33 ± 0.45, which was significantly lower than that of HT29 cells of 2.48 ± 0.85. The volume of the SW48 changes was significantly higher (about 3.5 times), after insertion of the cell body into the micropipette, when compared with HCT29 cells. SW48 cells have shown to be softer than the primary grade of CCCs, meaning that the decrease in cell elastic modulus was associated with their higher invasiveness. This property seemed to help the invasion of the cells through the microvasculature and cell spreading [51]. Extracellular vesicles (EVs) produced by the CRC cells must be a target, since according to Serrati et al. they contribute to the CRC dissemination into peritoneal cavities due to the EVs released, inducing the alteration of peritoneal mesothelial cells [202].

In terms of translation to the clinic, the AFM can be a promising tool in the surgery room, based on the concept that the CRC tissues are stiffer than healthy ones. In practice, after removing the tumor mass, the elasticity properties of tissues would be measured from the center to the periphery. When the stiffness values on the periphery of the tumor would be similar to the values of the healthy tissues around it, this would mean that it would not be necessary to extend the tumor area to be removed. Although this procedure is always carried out afterwards by anatomic pathology analysis, carrying out this analysis in situ would be faster and would avoid the eventual repetition of the surgery. A similar approach using Raman confocal microscopy has already been implemented in surgery of the head and neck in the Netherlands [203].

It was shown that, in CRC, using different approaches and techniques, bringing together biologists, physicists, biochemists, and medical specialists, is very important to deepen our knowledge and to develop novel drugs and treatments for CRC.

## 4. Conclusions and Assessment

Mechanotransduction has been shown to modulate cell functions in tumorigenesis and metastasis. Since the stiffness of the tumor microenvironment contributes to tumor growth and metastasis, trying to prevent or reverse the tumor stiffening could be an approach for new therapeutic strategies. Targeting the mechano-sensing mechanisms, through which cancer cells sense the stiffening of the tumor microenvironment, could be an option for developing anti-tumorigenic therapies. In CRC, the cytoskeleton reorganization of cells can be a good biomarker of cell state during cancer progression, providing crucial information for new developments in cancer diagnostics, therapies, and drug efficiency. Integrins must be explored since these molecules are crucial to transfer forces from the ECM through the cell membrane and cytoskeleton.

Several biomechanical and biochemical pathways were reviewed, showing how biophysical cell and ECM parameters have an impact on biochemical cell signaling. Wnt/β-catenin, YAP/TAZ, TNF-α, TGF-β, APC, Rho family, RAS, p53, and P13k are known pathways activated in CRC. Mechanical stimuli, such as matrix stiffness, matrix elongation, and cell density, influence the previous signaling pathways. The fate of epithelial cells in the intestine and colon is controlled by a dominant force coming from the Wnt/β-catenin cascade, as well as the mutation in the APC. Hippo signaling, through the YAP/TAZ, is related to cell–ECM adhesion sites, where it senses cell geometry and detects matrix rigidity. ECM-nuclear mechanical coupling translocates YAP in response to substrate rigidity. TNF-α is recognized as one of the major mediators of cancer-related inflammation, mediating all steps of tumorigenesis, such as proliferation, invasion, angiogenesis, and metastasis formation by accelerating tumor invasion and metastasis through EMT. Rho family activation is also related with cancer metastasis. The activation of these actin nucleators induces the formation of stress fibers, lamellipodia and filopodia. The invasion of cancer cells is associated with the formation of several actin-mediated structures, including lamellipodia, filopodia, podosomes, and invadopodia. RAS is implicated in ECM topography, stiffness, density, and porosity changes. The family of apoptosis-stimulating proteins of p53 is dysregulated in CRC patients. P13k dysregulation is related to the crypt buckling tissue and ECM alterations. TGF-β promotes the excess of ECM production and mediates the crosslinking of ECM through the upregulation of lysyl oxidase. The crosslinking of ECM increases its resistance when forces are applied, being translated in the increase of elasticity.

CAFs are among the cells that promote angiogenesis in CRC. In addition, they promote the disruption of the BM, facilitating the invasiveness of the CRC. CAFs also produce several MMPs, among which some are responsible for angiogenesis. CAFs inactivation should be further investigated. EMT is also an important process in CRC, being crucial to increase efforts in the investigation of substances to stop EMT.

Regarding intravasation into blood vessels, CRC cells are exposed to shear forces exerted by blood flow, which facilitates the interaction of cancer cells with endothelial cells, therefore promoting extravasation. New therapies should target the interaction between CRC and endothelial cells from vessel walls, impairing the bindings between the adhesion molecules of cancer cells and the vessel site receptors, contributing to decrease in extravasation and metastization.

High internal solid and fluid pressures of the tumor microenvironment contribute mostly to restricted drug delivery to tumor cells. Hypoxia contributes to solid tumors due to the compression of blood and lymphatic vessels and induces resistance to chemo-, radio-, and immunotherapies. Accordingly, therapeutic strategies that target the sources of high solid or fluid pressure in the tumor vasculature can greatly improve the main route of drug access through drug delivery to the tumor site.

It is expected that liquid biopsy techniques with circulating tumor cells from blood will become common practice to be used as a diagnostic routine for CRC as well as other cancers.

Additionally, 3D studies should become more widely used since they provide a similar environment to the native structure, thus providing a better and more meaningful translation of research to the clinics.

The translation of AFM to the clinical could be a huge advantage in the diagnosis of cancer for the distinction of healthy and tumor tissues, in situ during the surgery, through the determination of nanomechanics.

## Figures and Tables

**Table 1:** Characterization techniques to study the mechanobiology and mechanotransduction.

Technique	Principle	Refs.
Atomic force microscopy (AFM)	Mechanical characterization of samples in physiological conditions; allows quantification of mechanical properties of cells and tissue samples and also quantification of molecules and cells interactions by force spectroscopy, using dynamic conditions.	[82,112,113,114]
Microindentation	Quantification of micro-mechanical properties of biomaterials, hydrogels and biological samples down to cell-length scale, in physiological and dynamic conditions	[115]
Rheometry	Quantification of mechanical properties of soft tissues, thus being a promising technique for cancer diagnosis, as well as for assessment of effectiveness of anticancer treatments, in dynamic conditions	[116]
Shear wave elastography and magnetic resonance elastography	Characterization of healthy and diseased tissues in a non-invasive manner, with the generation of cross-sectional images, showing the stiffness of the tissue.	[117,118]
Elastography	Quantification of stiffness of the tissue is evaluated by the tissue response to an externally applied mechanical stimulus, leading to a measurable tissue deformation.	[119]
Micropippete aspiration	Allows study of a whole cell which, under the suction pressure, undergoes a deformation process, thereby allowing measurement of geometry of the cell inside the capillar.	[120]
Optical stretching	Formation of an optical trap, resulting from the two opposing laser beams, presenting a Gaussian intensity distribution, able to capture the cell that is in suspension and stretching it in situ; it acts on the entire cell membrane, making it possible to measure its viscoelastic properties.	[121]
Magnetic twisting cytometry	It is based in the application of a twisting field, generating rotational shear stresses in several directions; it allows the attachment of specific beads to the cytoskeleton through the membrane mechanoreceptors, applying local mechanical stress to live cells.	[122]
Terahertz waves	It is used in cancer diagnosis, not through mechanical properties but using imaging and spectroscopy (in a range of 0.1 and 10 THz).	[123,124]

**Table 2:** Biophysical cues and its relation with biochemical pathways, their influence on CRC behavior, prognosis, and possible therapeutic strategies.

Biophysical Cues	Biochemical Pathway Activation	Cell Process Activation	Prognosis	Therapeutic Target	Refs.
Cell stress (UV, hypoxia) ECM stiffness	p53	Loss of cell adhesion promoting EMT Stroma enriched in CAFs DNA damage Filopodia formation	Good	Promote the wild-type p53 Avoid CAFs differentiation Avoid the ECM stiffness Regulating the expression of E- and N-cadherins, p53 prevents EMT	[39,66,156,157]
ECM stiffness	APC (loss) APC (overexpression)	Decrease of microtubule stability Shape changes in cells Cell deformation Loss of cell adhesion Induce cell protrusions Alter cell shape Cellular asymmetry by inducing longer cellular protrusion	Good	Validating the Wnt pathway as a therapeutic target	[13,132,135,158]
Cell friction forces ECM rigidity Fluid shear stress Forces applied on the nucleus	Hippo ON (YAP/TAZ off) Hippo ON (YAP/TAZ off) Hippo OFF (YAP/TAZ on)	Cell size increment Cell size increment MMP-7 increasing (break the BM) Cell stretching and density increasing Β-catenin inside the nucleus More research must be performed	Poor	The therapy must be applied according to the CMS	[39,142,143,147,159,160,161,162,163,164]
ECM stiffness	KRAS	EMT Cellular plasticity PI3K activation Cytoskeletal deformability	Poor	Targeting EMT	[66]
ECM via interaction with fibronectin through tenascin-C ECM stiffness	Rho-family	Filopodia formation Lamellopodia formation Podosomes formation Contractability Activation of actin regulation Stress fibers increase Loss of cell polarity	Poor	Target the integrins and ECM connections; target the tenascin-C Targeting VEGF and EGFR	[39,50,141,153]
ECM stiffness Shear stress Compression Cell adhesion	Wnt (on) Wnt (off)	APC mutation activation Nucleus with β-catenin inside More studies are need	Good		[95]

Note: APC, Hippo, and Wnt signaling pathways have a dual role in CRC cells (two colors were used, corresponding to each case: blue—if the biochemical pathway is downregulated, loss or off; red—if the biochemical pathway is overexpressed or on).
